# Evaluation of trabecular changes following advancement genioplasty combined with or without bilateral sagittal split osteotomy by fractal analysis: a retrospective cohort study

**DOI:** 10.1186/s12903-023-02860-z

**Published:** 2023-03-18

**Authors:** Gökhan Çoban, Taner Öztürk, Süheyb Bilge, Emin Murat Canger, Ahmet Emin Demirbaş

**Affiliations:** 1 Department of Orthodontics, Faculty of Dentistry, Erciyes University, Kayseri, Türkiye; 2 Department of Oral and Maxillofacial Surgery, Faculty of Dentistry, Erciyes University, Kayseri, Türkiye; 3 Department of Oral and Maxillofacial Radiology, Faculty of Dentistry, Erciyes University, Kayseri, Türkiye

**Keywords:** Chin repositioning, Fractals, Genioplasty, Osteotomy, Sagittal split ramus

## Abstract

**Background:**

It is aimed to investigate whether there was a difference in radiographic changes in the operational areas between genioplasty alone and genioplasty combined with mandibular advancement and to evaluate the fractal dimension (FD) to assess trabecular changes after genioplasty surgery.

**Methods:**

Preoperative-(T0) and postoperative-(T1) panoramic radiographs of 26 patients without any complications who underwent genioplasty combined with bilateral sagittal osteotomy and mandibular advancement or genioplasty alone were selected. In the panoramic radiographs of both groups, the genial segment, mandibular angulus, and surgical osteotomy line were examined using FD. The box-counting method was used for FD evaluation.

**Results:**

It was determined that FD values before and after treatment were similar in both groups for all regions where measurements were made. After surgery, the FD values of the middle region of the genial segment were found to be significantly lower than the other regions. At T1, the FD values at the osteotomy area were found to be significantly higher than those in the middle region of the genial segment.

**Conclusion:**

Trabecular structure does not differ in patients undergoing genioplasty alone or in combination with mandibular advancement osteotomy. The middle region of the genial segment heals later than other regions.

## Background

Complex geometric structures consisting of curves, points, and surfaces that cannot be defined by shapes (such as square, triangle, or circle) that have the property of appearing independently of each other are called fractals [1, 2]. The method that expresses these structures numerically (fractal dimension; FD) and reveals the structure complexity is fractal analysis [3]. It has previously been described that FD increases with increasing complexity and decreases with decreasing complexity of an object or structure [4, 5]. Although there are various methods to calculate the fractal size of a complex structure with fractal analysis, which is easily accessible, simple to implement, and not affected by projection geometry or radiological features, currently, the most used method is the box-counting method [1, 3, 4]. Notably, the human body can be considered a fractal structure that is like itself and consists of complex structures such as bronchial tubes, blood vessels, brain folds, and bone structures [6–8]. In recent years, this method has become very common in the examination of bone structures in the medical field, and especially in dentistry, in which it is used to reveal the trabecular structure in the jaw bones and evaluate the changes [8, 9]. In the field of dentistry, FD examinations are frequently used in problems that cause changes or differences in the structure of the alveolar bone, such as periapical lesions, bruxism, implant surgery, temporomandibular joint dysfunctions, orthognathic surgical treatments, and periodontitis [4–6, 10–15]. The density of the trabecular bone, where metabolic activity can be observed more clearly compared to the cortical bone, and the arrangement of the trabeculae show the mechanical properties of the bone. As the trabeculae in the structure increases, the complexity increases, and FD values follow this [5–16].

Skeletal class II malocclusions, which are mostly characterized by the mandibular retrognathia, cause functional problems such as aesthetic, respiratory and occlusion, and can be treated with various appliances and surgical-orthodontic procedures [17, 18]. There are various (surgical or non-surgical) methods for the correction of skeletal structures in disharmonic patients that affect the dentofacial appearance and functions (breath, mastication etc.) [6, 12, 19–21]. In individuals who do not have mandibular asymmetry or position problems, genioplasty alone is applied to adapt the chin projection to the facial profile for aesthetic reasons [20]. Mandibular advancement with bilateral sagittal split osteotomy (BSSO) is applied to improve dentofacial aesthetics in adults with a skeletal class II relationship originating from mandibular retrognathia. In addition, depending on the patient’s aesthetic complaint, mandibular advancement with BSSO can be combined with genioplasty simultaneously [22]. Clinical signs, which are often subjective, are used to evaluate the bone healing process after orthognathic surgical treatment [12]. Radiography methods can be used for objective evaluation, as stated in a few studies [6, 12, 15]. In cases in which the genial area is taken forward with genioplasty, there is very little improvement in recovery. In the 1988 report by Storum et al. in which monkeys were studied, it was shown that an improvement in the osteotomy line started in the fourth week [23]. Gianni et al., on the other hand, examined the neurosensory change in individuals who had only genioplasty or had genioplasty combined with BSSO and reported that the combined application had a more negative effect on recovery [24]. However, there are still uncertainties about bone healing.

The aim of this study is to examine the radiographic changes in the cancellous bone in the osteotomy line of the genial region (chin) after the genioplasty procedure, which is surgically taken forward in different vertical dimensions with or without mandibular orthognathic surgery (BSSO), and to evaluate them quantitatively by fractal analysis to compare them with other regions of the mandible.

## Materials and methods

### Sample

This retrospective cohort study was approved by the Erciyes University Clinical Research Ethics Committee prior to initiation (Approval code: 2020/485; Date: 23/09/2020). Panoramic and cephalometric radiographs with treatment information data from 26 patients who underwent genioplasty for chin correction at the Erciyes University Faculty of Dentistry between January 2016 and January 2020 (18 women (29.94 ± 5.82 years) and 8 men (31.37 ± 11.90 years)) were included in the study (Table 1).

**Table 1:** Demographic characteristics

	Female [N (%)]	Male [N (%)]	Total [N (%)]
Gender	18 (69.2)	8 (30.8)	26 (100.0)
Mean Age (year)	29.94 + 5.82	31.37 + 11.90	28.31 + 8.18
Movement Types	Only Forward [N (%)]	Forward and Upward [N (%)]	Forward and Downward [N (%)]
Total	8 (30.8)	12 (46.2)	6 (23.1)
Vertical Movement (mm)	-	4.00 ± 1.54	3.83 ± 1.94
Sagittal Movement (mm)	4.79 ± 1.51	2.75 ± 2.14	2.42 ± 1.91

* Data was presented mean ± SD. mm: Millimeter. N: Number of subjects

Inclusion criteria were 1- absence of a congenital or developmental craniofacial anomaly or syndrome, 2- patients with pre- and postoperative cephalometric and panoramic radiographs with adequate radiographic quality, 3- adult patients over 18 years of age, and 4- patients who recovered normally without any complications or reoperation after genioplasty. Individuals who had previously undergone genioplasty, had any traffic accident or traumatic injury to the face, or had systemic disease were excluded from the study. Of the 26 patients included in the study, 14 (53.8%) had genioplasty only and 12 (46.2%) had simultaneous mandibular advancement with BSSO combined with genioplasty. The individuals who underwent mandibular advancement with BSSO were adults with a skeletal class II relationship originating from mandibular retrognathia, and they requested genioplasty for aesthetic reasons.

### Surgical procedure for genioplasty

The mucoperiosteal flap was reflected with a cautery incision along the labial sulcus, 5 mm below the keratinized gingiva, between the mandibular right and left canines. Dissection was performed so that the bilateral mental nerve was identified and protected, and the chin was exposed. The planned osteotomy line, 5 mm inferior to the mental foramen and apical to the mandibular anterior teeth, was marked horizontally with a pencil. As planned, osteotomy was performed with an ultrasonic piezo saw and the genial segment was mobilized. The genial segment was repositioned forwardly and downwardly or upwardly, and the defect was grafted with a 5 mm wide otology block graft. Grafts were used in only 6 cases where the separated genial segment was taken forward and downwards. The genial segment was fixed in its new position with a pre-bended 5-hole genioplasty plate and mini screws specially designed for the genioplasty operation, bringing the chin tip forward by average 7 mm [25]. A 30*40 mm collagen membrane was covered on the osteotomy line and graft. First, the mental muscle was sutured with a 4/0 maxon. The connective tissue and mucosa were then closed with 4/0 vicryl.

### Radiological analysis

#### Measuring the amount of movement of the genial segment on lateral cephalometric radiographs

Lateral cephalometric radiographs taken from individuals for cephalometric examination were performed by the same technician using the same device (Orthoceph OP300, Instrumentarium, Tuusula, Finland) with the Frankfort horizontal plane parallel to the ground. All cephalometric radiographs were evaluated in Dolphin Imaging Software (version 11.3; Dolphin Imaging and Management Solutions, Chatsworth, CA, USA). Measurements were made according to the true horizontal plane drawn 7° to the sella-nasion line in the cephalometric radiograph and the true vertical plane passing through the sella perpendicular to this plane [26]. The perpendicular lengths of the pogonion point to these planes were measured before and after surgery [27]. Analyses of all radiographs were performed on the same day by the same investigator.

#### Fractal analysis

**Fig. 1:**
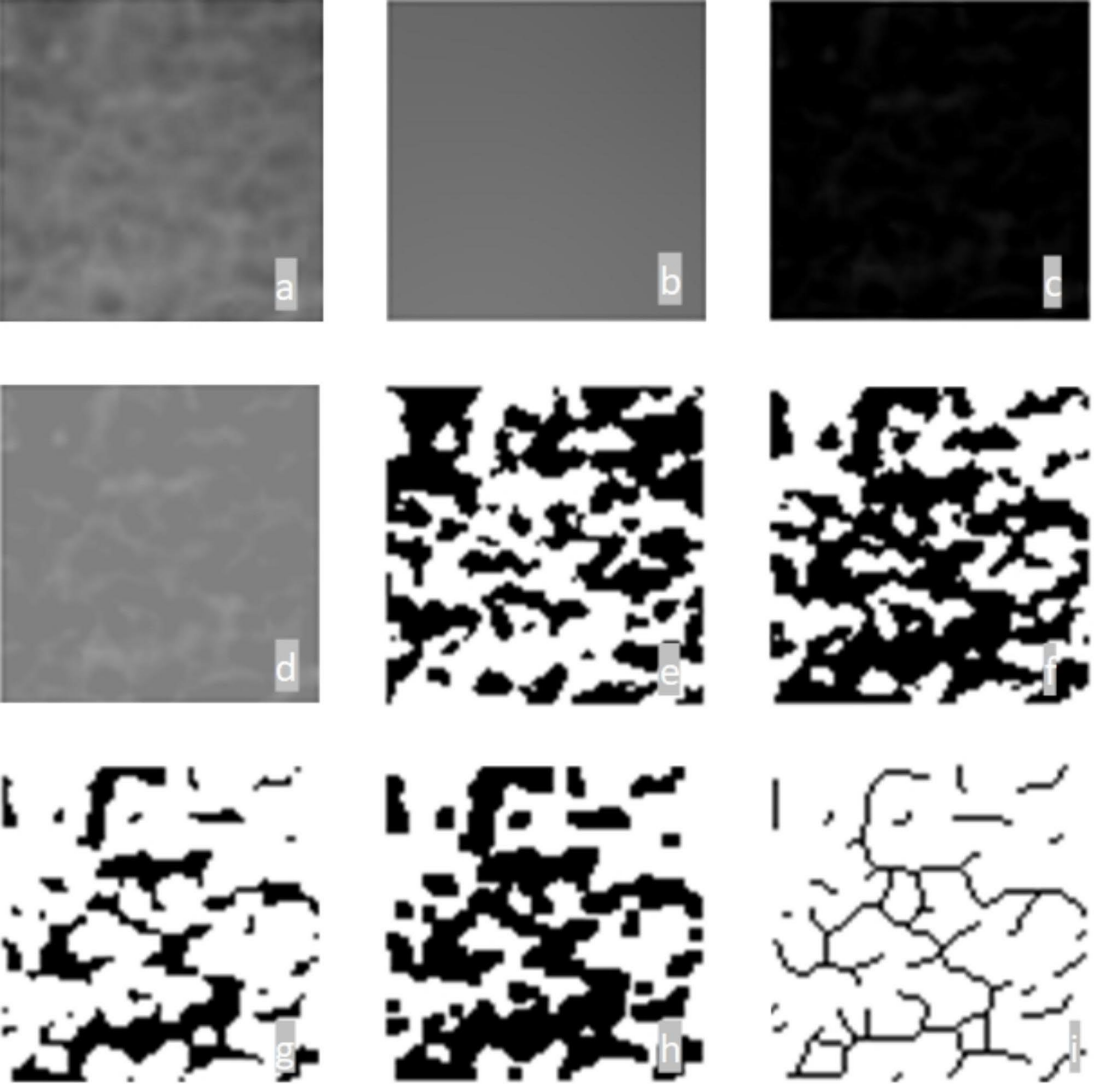
Sequential radiographical presentation of the fractal analysis with box-counting method. (A) Cropped and duplicated region of interest. (B) Gaussian blurred image. (C) Subtraction of the blurred images from the original cropped image. (D) 128 pixel added version of the image in figure C. (E) Binarized version of the image in figure D. (F) Inverted version of the image in figure E. (G) Eroded version of the image in figure D. (H) Dilatated version of the image in figure G. (I) Skeletonized version of the image in figure G

**Fig. 2:**
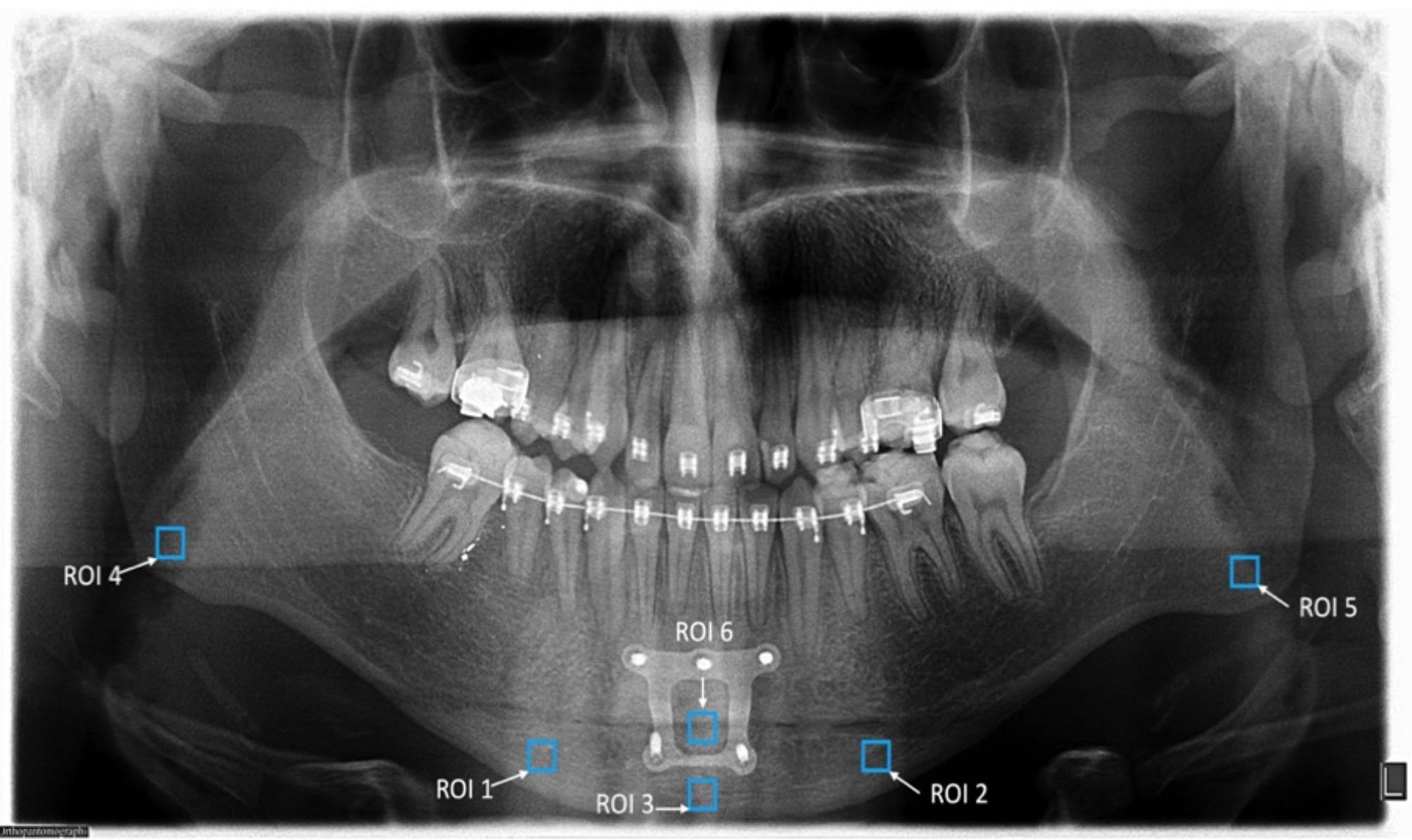
The region of interests (ROIs) was obtained from the panoramic radiographs, a square of 50 × 50 pixels. ROI 1: right region of the separated genial segment. ROI 2: left region of the separated genial segment. ROI 3: middle region of the separated genial segment. ROI 4: region selected in the right mandibular angulus. ROI 5: selected region in left mandibular angulus. ROI 6: healing region between the separated genial segment and mandibular corpus

All panoramic radiographs used for evaluation were taken with the same equipment (OP200 D; Instrumentarium Dental, Tuusula, Finland; 66–85 kVp, 10–16 mA, 14.1 s exposure time) with the same specifications and by the same technician. The patients were positioned in accordance with the recommendations of the device manufacturer, with the Frankfort horizontal plane parallel to the ground and the sagittal plane aligned with the vertical line on the device. Radiography images were exported in TIFF format with a 2976 × 1536 pixel size and 5.5 LP/mm resolution. All images were analyzed by the same researcher using a 32” Dell LCD screen with a resolution of 1280 × 1024 pixels in a dark room and ImageJ image analysis software (version 1.3; National Institutes of Health, Bethesda, MD, USA) using a Dell Precision T5400 workstation (Dell, TX, USA). Many methods have been used to calculate FD [8]. In this study, the box-counting method recommended by White and Rudolph, which is frequently used in the literature, was used [28]. The steps generally applied in the box-counting method involve plotting on a logarithmic scale with a line drawn according to the values obtained. The slope of the drawn line gives the FD of the structure [3, 13]. In methods that calculate FD based on distance measurement, an edge length of the pixel is used as the unit of length. In methods that calculate the FD according to the volume measurement, the perimeter of the pixel is used as the volume unit. In this method [13], circles of various diameters are randomly placed in the image and the pixels belonging to the image border inside the circles are counted (Fig. 1). Radiographs from patients who underwent panoramic radiographs before surgery and 6 months after surgery were selected. The measurements were obtained from the panoramic radiographs, a square of 50 × 50 pixels: two regions of interest (ROIs) were selected bilaterally on the mandibular angulus area, three ROIs were selected within the genial segment of the mandible, and one ROI was selected on the osteotomy healing area on both radiographs (Fig. 2) [29]. In the present study, the reason for evaluating the angulus regions (ROI 4 and 5) that will not be affected by occlusal forces and surgical procedures is that the regions that are not included in the osteotomy area were also examined for individual control in the literature [4, 29, 30]. In order to ensure the reproducibility of the measurements, except for the osteotomy line measurement, the other measurements were made to be at the same level of the teeth and just above the cortical bone border and at the same size as the first measurements. All radiographic analysis was performed by a dentomaxillofacial radiologist (E.M.C.) with 15 years of experience.

### Sample size calculation

The sample size calculation for this study was based on the data obtained from the study by Kang et al. in which the changes in the mandibular bone structure before and after orthognathic surgery were examined by FD [7]. It was determined that using a total of 12 samples would be sufficient for this study, according to the results of two-way paired samples t-test power analysis performed using G*Power software (ver. 3.1.9.7, Heinrich Heine University, Duesseldorf, Germany) at 85% power, an alpha level of 0.05, and an effect size (d) of 1.00.

### Statistical analysis

The Sigma Stat software (ver. 3.5, Systat Software, Point Richmond, CA, USA) was used in the statistical evaluation of the data. The Shapiro–Wilk test was used to evaluate the normality of the data. Normally distributed data were evaluated using the paired samples t-test for pairwise evaluations between dependent groups and the Wilcoxon signed-rank test was used for non-normally distributed data. In the evaluation of repeated measurements of the same region at the same time, the Friedman Repeated Measures Analysis of Variance on Ranks test was used for non-normally distributed data. For measurements for which significant differences were revealed in these tests, the Student–Newman–Keuls method was used as a post hoc test in paired comparisons. The Spearman correlation coefficient was used to examine the correlation between the amount of genial segment movement and the FDs of an ROI. For all the tests, the statistical significance level was set at P < 0.05.

### Reliability analysis

In order to evaluate the intra-observation reliability, 6 of the samples randomly selected by the same researcher were re-performed at least 1 month after the first measurement. Intraclass correlation coefficient (ICC) was used to evaluate the reliability between the two measurements, and the Cronbach-α coefficient was determined as 0.992 (Lower bound 0.905; Upper bound: 0.998). With this result, the measurement reliability was determined as very strong [31].

## Results

There was an average of 7 months (7.77 ± 1.34 months; range: 6–12 months) between radiographs before (T0) and after (T1) surgery. Since there was no significant difference between the FD values of male and female individuals included in the study, all samples were evaluated as a single group. Patients were classified as anterior only forward (n = 8; 30.8%), forward and upward (n = 12; 46.2%), and forward and downward (n = 6; 23.2%) according to the movement performed in the genial segment during genioplasty, and it was determined that there was a significant difference between the movement type groups in terms of sagittal movement amounts of the genial segment. Notably, the amount of movement in the only forward group (4.79 ± 1.51 mm) was significantly higher than in the forward and upward group (2.75 ± 2.14 mm; P < 0.05; Table 1). However, FD values did not differ between these movement groups. The FD values of these groups did not differ significantly before and after treatment, nor did the difference between the two measurements differ between groups (P > 0.05; Table 2). Therefore, all individuals were evaluated as a single group and a comparison before (T0) and after (T1) treatment was made.

**Table 2:** Comparison of the FD values between the groups before, after the treatment and difference between the time-points

		Only Genioplasty (n = 14)	MABSSO combined with Genioplasty (n = 12)	P values*
T0	ROI 1	1.445 ± 0.050	1.483 ± 0.072	0.124
	ROI 2	1.426 ± 0.052	1.408 ± 0.054	0.397
	ROI 3	1.455 ± 0.062	1.444 ± 0.094	0.733
	ROI 4	1.451 ± 0.063	1.433 ± 0.078	0.681
	ROI 5	1.450 ± 0.056	1.452 ± 0.063	0.921
T1	ROI 1	1.459 ± 0.058	1.436 ± 0.072	0.376
	ROI 2	1.405 ± 0.088	1.365 ± 0.086	0.090
	ROI 3	1.425 ± 0.058	1.447 ± 0.050	0.315
	ROI 4	1.491 ± 0.057	1.443 ± 0.065	0.057
	ROI 5	1.468 ± 0.054	1.452 ± 0.082	0.562
	ROI 6	1.427 ± 0.082	1.416 ± 0.052	0.687
Diff.	ROI 1	0.014 ± 0.066	-0.047 ± 0.081	0.051
	ROI 2	-0.021 ± 0.105	-0.043 ± 0.113	0.681
	ROI 3	-0.029 ± 0.085	0.003 ± 0.106	0.385
	ROI 4	0.039 ± 0.072	0.009 ± 0.081	0.381
	ROI 5	0.019 ± 0.058	0.001 ± 0.052	0.535
Duration of 2 radiographic evoluation (month)		8.18 ± 5.44	6.33 ± 2.31	0.498

Data was given Mean ± Standard Deviation. ROI: Region of Interest. MABSSO: Mandibular Advancement with Bilateral Sagittal Split Osteotomy. T0: Pre-treatment values. T1: Post-treatment values. Diff.: Values of T1 to T0 Difference

ROI 1: right region of the separated genial segment. ROI 2: left region of the separated genial segment. ROI 3: middle region of the separated genial segment. ROI 5: region selected in the right mandibular angulus. ROI 5: selected region in left mandibular angulus. ROI 6: healing region between the separated genial segment and mandibular corpus. * Results of Independent Samples-t test

Comparison of the FD changes that occurred with the genioplasty application is presented in Table 3. When the change between T0 and T1 was examined, it was determined that there was no significant difference for any ROI values (Table 3). However, when the ROI values were examined within everyone, no difference was observed at T0, but significant differences were found in the change at T1 (P < 0.001) and differences between T0 and T1 (P = 0.030). The FD values of ROI 2 performed in the middle region of the genial segment were found to be significantly lower than the other regions after treatment (P < 0.05). When the changes in the measurement regions were examined, it was determined that the FD values in the right region of the genial segment were like those of the right and left angulus and significantly higher than in the genial middle and left regions (P < 0.05).

**Table 3:** Comparison of FD values before (T0) and after (T1) treatment and between measurement regions

N= (26)	T0		T1		Diff.		P values
	Mean ± SD	Median (25% / 75%)	Mean ± SD	Median (25% / 75%)	Mean ± SD	Median (25% / 75%)	
ROI 1	1.462 ± 0.063	1.471 (1.410–1.509)	1.448 ± 0.064 ^a^	1.450 (1.413/1.500)	0.030 ± 0.074 ^a^	0.040 (-0.030/0.095)	0.382 ^Pt^
ROI 2	1.418 ± 0.053	1.413 (1.392/1.447)	1.386 ± 0.089 ^b^	1.392 (1.360/1.438)	-0.031 ± 0.107 ^b^	-0.024 (-0.054/0.043)	0.334 ^Wr^
ROI 3	1.450 ± 0.077	1.466 (1.405/1.491)	1.435 ± 0.055 ^a^	1.446 (1.392/1.472)	-0.014 ± 0.094 ^b^	-0.014 (-0.097/0.062)	0.450 ^Pt^
ROI 4	1.443 ± 0.069	1.454 (1.399/1.503)	1.469 ± 0.064 ^a^	1.472 (1.425/1.525)	0.026 ± 0.076 ^a,b^	0.013 (-0.026/0.078)	0.093 ^Pt^
ROI 5	1.451 ± 0.058	1.433 (1.409/1.512)	1.460 ± 0.068 ^a^	1.474 (1.421–1.510)	0.010 ± 0.056 ^a,b^	0.002 (-0.014/0.038)	0.379 ^Pt^
P values	0.146 (F = 1.744) *		P < 0.001 (F = 6.849) *^,^ ***		0.030 (Chi-square = 10.674) **^,^ ***		

SD: Standard Deviation. ROI: Region of Interest. T0: Pre-treatment values. T1: Post-treatment values. Diff.: Values of T1 to T0 Difference

ROI 1: right region of the separated genial segment. ROI 2: left region of the separated genial segment. ROI 3: middle region of the separated genial segment. ROI 5: region selected in the right mandibular angulus. ROI 5: selected region in left mandibular angulus

^Pt^: Result of Paired-Samples-t test. ^Wr^: Result of Wilcoxon Signed Rank test. * Result of One Way Repeated Measures Analysis of Variance test. ** Result of Friedman Repeated Measures Analysis of Variance on Ranks test. *** Results of All Pairwise Multiple Comparison Procedures (Student-Newman-Keuls Method). The same letters in the column indicate that there is no difference in pairwise comparison

The comparison of the FD values of the measurement regions in the genial osteotomy line, including the healing line, taken after the treatment is provided in Table 4. In the evaluation, it was found that the lowest FD values were in the middle genial segment (1.386 ± 0.089), followed by the genial osteotomy line (1.422 ± 0.068), and it was determined that other regions had significantly higher FD values than these two regions (P < 0.001). There was no significant correlation between the amount of sagittal and vertical movement of the genial segment and the FD values of the genial segment regions and the genial osteotomy line (Table 5).

**Table 4:** Comparison of the measurement regions among themselves in the post-treatment (T1) evaluation

N= (26)	Mean ± SD	Median (25% / 75%)
ROI 1	1.448 ± 0.064 ^a^	1.450 (1.413/1.500)
ROI 2	1.386 ± 0.089 ^b^	1.392 (1.360/1.438)
ROI 3	1.435 ± 0.055 ^a^	1.446 (1.392/1.472)
ROI 4	1.469 ± 0.064 ^a^	1.472 (1.425/1.525)
ROI 5	1.460 ± 0.068 ^a^	1.474 (1.421–1.510)
ROI 6 ^†^	1.422 ± 0.068 ^c^	1.419 (1.391/1.474)
P value *^,^ **	P < 0.001 (Chi-Square = 21.385)	

SD: Standard Deviation. ROI: Region of Interest

ROI 1: right region of the separated genial segment. ROI 2: left region of the separated genial segment. ROI 3: middle region of the separated genial segment. ROI 4: region selected in the right mandibular angulus. ROI 5: selected region in left mandibular angulus. ROI 6: healing area between the separated genial segment and mandibular corpus

^†^ This ROI measurement was performed only to evaluate the healing of the area between the mandibular corpus and genial segment at the time-point of the T1. * Result of Friedman Repeated Measures Analysis of Variance on Ranks test. ** Results of All Pairwise Multiple Comparison Procedures (Student-Newman-Keuls Method). The same letters in the column indicate that there is no difference in pairwise comparison

**Table 5:** Investigation of the correlation between the genial segment movement amounts and the FD in the measured areas

	ROI 1	ROI 2	ROI 3	ROI 4	ROI 5	ROI 6
Advancement	-0.170	-0.116	-0.116	-0.134	-0.051	-0.165
Vertical Movement	0.022	0.063	0.066	0.322	0.235	0.080

* Data was presented as Spearman Correlation Coefficients. ROI: Region of Interest

ROI 1: right region of the separated genial segment. ROI 2: left region of the separated genial segment. ROI 3: middle region of the separated genial segment. ROI 4: region selected in the right mandibular angulus. ROI 5: selected region in left mandibular angulus. ROI 6: healing area between the separated genial segment and mandibular corpus

## Discussion

Bone healing after orthognathic surgery has been examined radiographically in a few studies in the literature [6, 12, 15]. These studies evaluated recovery after the BSSO procedure for mandibular prognathism. However, to our knowledge, there are no studies examining trabecular changes after genioplasty alone and/or genioplasty combined with BSSO. In this study, trabecular changes after genioplasty advancing the genial segment only and genioplasty combined with BSSO and mandibular advancement was evaluated and compared.

In many studies in the literature in recent years, it has been shown that changes in bone structure can be examined with FD in conditions affecting bone structure such as osteoporosis, hyperparathyroidism, osteoarthritis or osteogenesis imperfecta [9, 16, 32, 33]. In these studies, the differences in bone structure between healthy and sick individuals, as well as the effectiveness of treatments applied to sick individuals in some of the studies, were examined by comparing the changes in FD values occurring in the bone structure. Such studies have revealed that FD values can show differences in bone structure. Today, a wide variety of applications (etc. stem cell) are used to support the healing of bone structure during the treatment of changes caused by diseases [34]. It is often not possible to evaluate the success of them with methods that are less invasive than histological methods. However, FD analysis, which is evaluated on radiographs, comes to the fore in this regard. In the field of dentistry, it has been frequently used for detection of pathologies such as temporomandibular diseases, periodontitis and bruxism, and to examine the differences and changes in bone structure after therapeutic applications such as root canal treatment, implant surgery, orthognathic surgery, extraction of third molar or palatal expansion [4–6, 10, 11, 14, 35, 36]. In these studies, it has been reported that dental treatments can cause changes in the alveolar bone structure and that FD analysis can be used in the evaluation of treatment results. Cone-beam computed tomography (CBCT) can give more detailed and clear results, but it causes higher levels of radiation compared to panoramic radiographs in periodic examinations [37]. In the study by Magat and Ozcan Sener, it was stated that in the evaluation of the trabecular structure of the alveolar bone, it is appropriate to use panoramic radiography instead of CBCT, which has low image resolution and high overall radiation dose [38]. Therefore, panoramic radiographs were used in this study. In the literature, there are studies examining the recovery after implant surgery with fractal analysis [14]. When the panoramic radiographs taken before and after implant surgery were analyzed with fractal analysis, it was found that the increase in the fractal size of the bone around the implant correlated with successful osteointegration and healing of the trabecular bone [14]. In addition to implant surgery, recovery stages in orthognathic surgery cases can be followed with FD. Heo et al. published a study of 35 patients with the diagnosis of mandibular prognathism who were scheduled for BSSO [12]. They reported that no significant difference could be detected in the FD values before and one year after surgery. Moreover, they stated that it would be more accurate to examine trabecular changes with FD, which is an objective and quantitative method, compared to visual diagnosis, which is a subjective observation [12]. In the study by Kang et al., it was reported that there was a decrease in the FD values of the bone in the molar and canine teeth region one month after the BSSO and mandibular set-back procedure to treat mandibular prognathism, and that this may accelerate orthodontic tooth movement [6]. In the study by Park et al., in which mandibular prognathism was treated with mandibular set-back using BSSO, the change in mandibular cortical bone thickness was examined with fractal analysis [15]. Accordingly, it was reported that the FD values of the cortical bone were low even at the sixth month after surgery, but the interdental bone did not show any difference. No difference was reported in patients undergoing genioplasty in this study. Moreover, the results of the present study are also consistent with the literature by Çolak et al., that bone healing in horizontal osteotomies at 6 months after orthognathic surgery was similar [29]. In contrast, it was concluded in the present study that genioplasty only and genioplasty combined with BSSO and mandibular advancement did not differ in radiographs taken at least 6 months later. However, even after at least six months after genioplasty alone and/or combined with BSSO, FD values did not yet reach normal in the healing line or in the middle region of the genial segment. In line with this study, adequate stability at 6 months after advancement genioplasty alone or combined with BSSO has been reported in previous study [39], but no data on recovery were found. Findings from this study support the assumption that this stability will be promoted by bone healing after an average of 6 months.

### Limitations

A limitation of the study is that panoramic radiographs that offer two-dimensional observation were used instead of CBCT, which offers three-dimensional observation. However, given that periodic observations were made, using two-dimensional observation allowed less radiation to be applied, and bone healing could be studied successfully. Moreover, in the study, only data related to advancement surgery are presented. Nonetheless, this study fills a gap in the literature. It may be recommended to compare different surgical procedures with a larger sample size in future studies.

## Conclusion

In conclusion, genioplasty alone and combined with BSSO with mandibular advancement do not differ in terms of recovery at least 6 months after surgery. After the surgical procedure, trabecular structure improvement of the middle region of the genial segment is late complete compared to other regions. Likewise, trabecular changes of the genial segment mid-area and surgical osteotomy line after the surgical procedure is late complete compared to other parts of the segment and non-surgical areas. There is no significant relationship between the amount of movement of the genial segment in the sagittal and/or vertical dimensions between bone healings. In cases where histological evaluation is not possible with the obtained results, FD assessment may be useful to evaluate bone healing in addition to clinical and cephalometric radiographic evaluations. Before more detailed analysis, it was foreseen that this method would make an additional contribution to the evaluations.

## Data Availability

The data that support the findings of this study are available from the corresponding author upon reasonable request.

## Abbreviations

FD: Fractal Dimension
BSSO: Bilateral Sagittal Split Osteotomy
mm: millimeter
kVp: kilovoltage peak
mA: Milli-ampere
ver.: version
ROI: Region of Interest
TIFF: Tag Image File Format
LCD: Liquid Crystal Display
CBCT: Cone-Beam Computed Tomography.
